# Lysosomal membrane integrity in fibroblasts derived from patients with Gaucher disease

**DOI:** 10.1247/csf.23066

**Published:** 2023-12-09

**Authors:** Asuka Hamamoto, Natsuki Kita, Siddabasave Gowda B. Gowda, Hiroyuki Takatsu, Kazuhisa Nakayama, Makoto Arita, Shu-Ping Hui, Hye-Won Shin

**Affiliations:** 1 Graduate School of Pharmaceutical Science, Kyoto University, Kyoto 606-8501, Japan; 2 Faculty of Health Sciences, Hokkaido University, Sapporo, Hokkaido 060-0812, Japan; 3 Graduate School of Global Food Resources, Hokkaido University, Sapporo, Hokkaido 060-0809, Japan; 4 Laboratory for Metabolomics, RIKEN Center of Integrative Medical Sciences, Yokohama 230-0045, Japan

**Keywords:** glucosylceramide, lysosome, Gaucher disease, lysosomotropic agent

## Abstract

Gaucher disease (GD) is a recessively inherited lysosomal storage disorder characterized by a deficiency of lysosomal glucocerebrosidase (GBA1). This deficiency results in the accumulation of its substrate, glucosylceramide (GlcCer), within lysosomes. Here, we investigated lysosomal abnormalities in fibroblasts derived from patients with GD. It is noteworthy that the cellular distribution of lysosomes and lysosomal proteolytic activity remained largely unaffected in GD fibroblasts. However, we found that lysosomal membranes of GD fibroblasts were susceptible to damage when exposed to a lysosomotropic agent. Moreover, the susceptibility of lysosomal membranes to a lysosomotropic agent could be partly restored by exogenous expression of wild-type GBA1. Here, we report that the lysosomal membrane integrity is altered in GD fibroblasts, but lysosomal distribution and proteolytic activity is not significantly altered.

## Introduction

Lysosomal storage disorders (LSDs) are a collection of inherited diseases primarily characterized by deficiencies of lysosomal enzymes, accompanying the accumulation of undegraded substrates in lysosomes (Farfel-Becker *et al.*, 2011; Horowitz and Zimran, 1994; Mistry *et al.*, 2017; Platt, 2014; Sun, 2018). Gaucher disease (GD) is the most common LSD and is caused by mutations in the lysosomal glucocerebrosidase gene *GBA1*, resulting in the deficiency of enzyme activity; lysosomal glucocerebrosidase normally hydrolyzes the glucose moiety from glucosylceramide (GlcCer) in lysosomes (Brady *et al.*, 1965; Stirnemann *et al.*, 2017). The consequences of this deficiency are generally attributed to the accumulation of the substrate GlcCer, particularly in macrophages, and cells in the bone marrow, liver, and spleen (Stirnemann *et al.*, 2017). The phenotypes of GD are variable and classified into three clinical forms. Patients with type I GD have primarily visceral symptoms and no neurologic symptoms. In contrast, types II and III are associated with neurological impairment. Patients with type I GD and carriers of *GBA1* mutations are predisposed to developing Parkinson’s disease (PD) (Do *et al.*, 2019; Sidransky *et al.*, 2009).

Fibroblasts derived from patients with GD has been used as a genetic model to characterize the alteration of cellular processes and used to obtain different cell types, such as macrophages and neurons, by generation of induced pluripotent stem cells (Arevalo *et al.*, 2022). However, the lysosomal distribution and lysosomal activity were not carefully investigated in GD fibroblasts. Moreover, conflicting results have been reported regarding the increase in intracellular GlcCer in GD fibroblasts. Some studies have indicated that the cellular GlcCer levels are increased in GD fibroblasts (Fuller *et al.*, 2008; Zama *et al.*, 2009). However, other studies have reported that the GlcCer levels remain unchanged (Saito and Rosenberg, 1985; Sasagasako *et al.*, 1994; Sun *et al.*, 2015). In this study, we investigated cellular distribution of lysosomes, lysosomal proteolytic activity, lysosomal membrane integrity and cellular GlcCer levels in fibroblasts derived from patients with GD.

## Results

### Lysosomal morphology and function are unaffected in fibroblasts derived from patients with GD

**Fig. 1:**
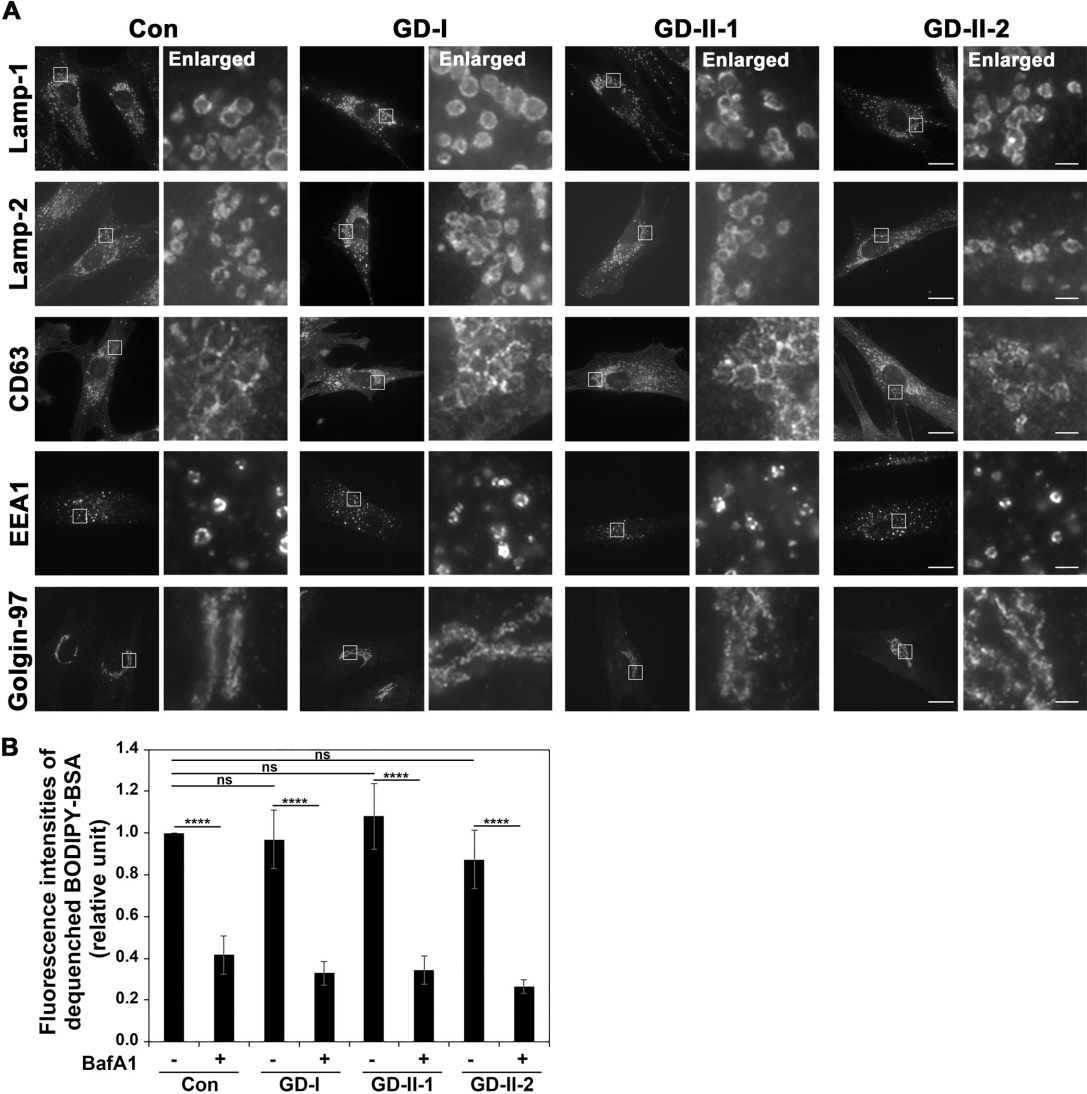
Cellular distribution and hydrolytic activity of lysosomes in GD fibroblasts (A) Normal fibroblasts (Con) and fibroblasts derived from patients with GD (GD-I, GD-II-1, and GD-II-2) were fixed and stained with primary antibodies against Lamp-1, Lamp-2, and CD63 (lysosomes), EEA1 (early endosomes), and Golgin-97 (Golgi complex), and then incubated with an Alexa Fluor 488-conjugated anti-mouse secondary antibody. Bars, 20 μm. The boxed regions were enlarged and shown on the right side (bars, 2 μm). (B) Cells were treated with the Self-Quenched BODIPY-BSA for 3 hr in the presence and absence of BafA1 (100 nM). Dequenched BODIPY signals in cells were measured by flow cytometry. The graph displays the average ± SD from four independent experiments. A one-way ANOVA was performed to assess variance, and comparisons were performed using Tukey’s post-hoc analysis. ****p<0.0001. ns, not significant.

GD is caused by the deficient enzymatic activity of lysosomal glucocerebrosidase, which is encoded by *GBA1* and removes the glucose moiety of GlcCer (Brady *et al.*, 1965; Stirnemann *et al.*, 2017). Thus, it is believed that GlcCer and/or its derivatives accumulate in lysosomes of GD cells (Sun, 2018). We firstly examined the distribution and morphology of subcellular organelles using several organelle markers (Fig. 1A) in healthy fibroblasts (Con) and fibroblasts derived from three patients with GD; one patient with type I GD [GD-I (L444P/L444P)] and two patients with type II GD [GD-II-1 (L444P/P415R) and GD-II-2 (L444P;E326K/L444P;E326K)](Sun *et al.*, 2015). As lysosomal markers, Lamp-1 and Lamp-2, lysosomal-associated integral membrane proteins with a single transmembrane domain, and CD63, a lysosomal tetraspanin protein, were investigated. Neither the distribution nor the morphology of these lysosomal protein-positive compartments was significantly affected in GD fibroblasts (GD-I, GD-II-1, and GD II-2) compared with healthy fibroblasts (Con). The morphology of other subcellular organelles, such as early endosomes (EEA1) and the Golgi complex (Golgin-97), was also unaffected in GD fibroblasts (Fig. 1A).

We next explored lysosomal function in these cells. We investigated proteolytic activity in lysosomes using BODIPY-labeled bovine serum albumin (BSA). Although the fluorescent signal of BODIPY is self-quenched due to fluorescence energy transfer between neighboring BODIPY molecules, BODIPY fluorescence is dequenched when BODIPY-BSA is endocytosed and BSA is degraded in lysosomes. The cellular fluorescent signal of BODIPY was measured by flow cytometry. After internalization of BODIPY-BSA, the BODIPY signal was detected in control cells, but was dramatically decreased by treatment with bafilomycin A1 (BafA1), which impairs lysosomal function by inhibiting vacuolar H^+^-ATPase (Fig. 1B). Somewhat unexpectedly, the BODIPY signal in GD fibroblasts was similar to that in control cells and was dramatically decreased by BafA1 treatment. This result indicates that proteolytic activity in lysosomes is not affected in GD fibroblasts. Taken together, these findings demonstrate that lysosomal morphology and function, at least the ability to degrade proteins, are not affected in GD fibroblasts. Consistent with our findings, it recently has been reported that lysosomal acidity and its proteolytic activity remain unchanged in GD fibroblasts (Patel *et al.*, 2023).

### Treatment with a lysosomotropic agent readily damages lysosomal membranes of GD cells

**Fig. 2:**
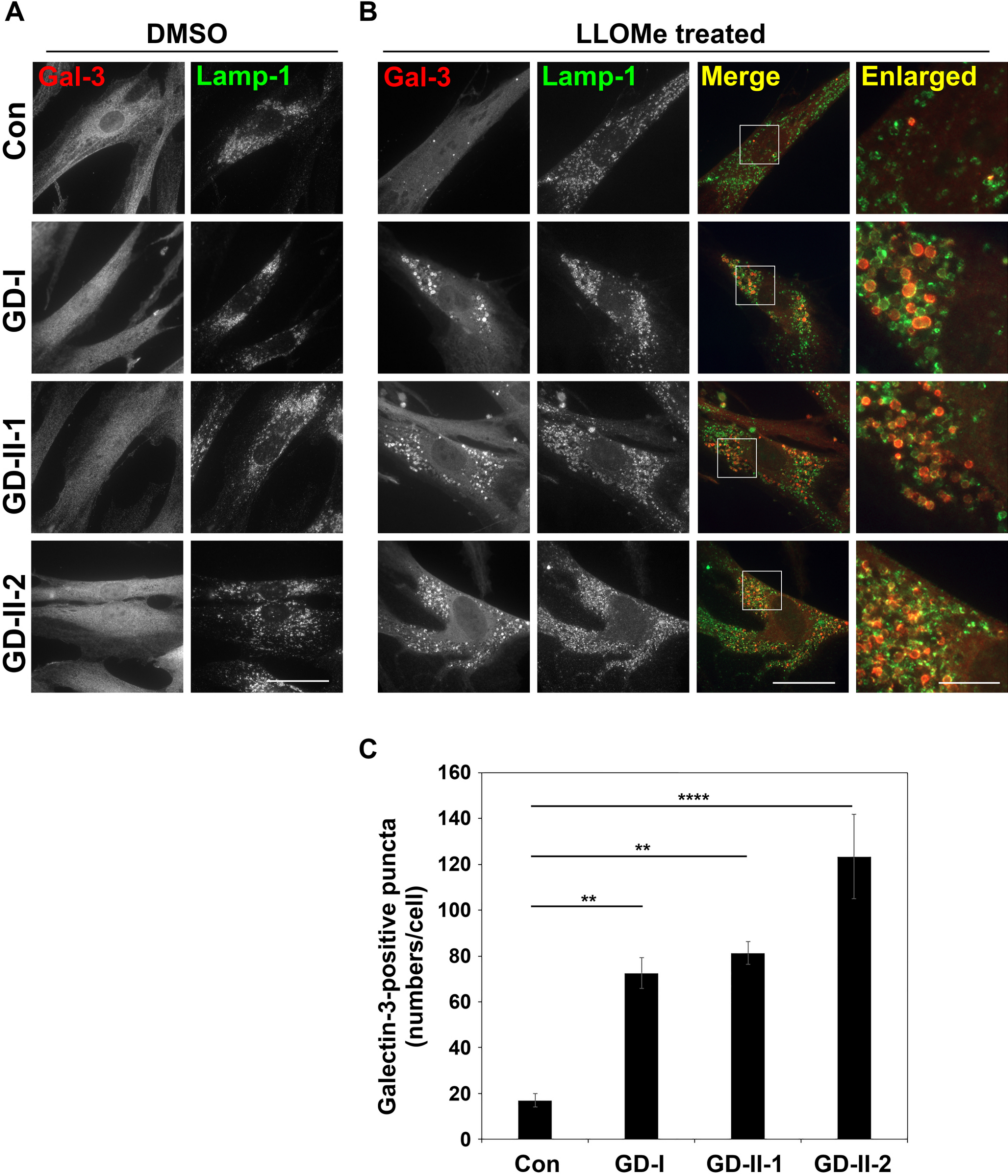
Lysosomal membrane integrity in GD cells Normal (Con) and GD fibroblasts were treated with DMSO (Mock) (A) or LLOMe (0.5 mM) (B, C) for 15 min. Cells were then fixed, incubated sequentially with primary antibodies against Gal-3 and Lamp-1, and with Cy3-conjugated anti-rat and Alexa Fluor 488-conjugated anti-mouse secondary antibodies. Bars, 40 μm. The boxed regions were enlarged and shown on the right side (bars, 10 μm). (C) The number of Gal-3-positive puncta per cell was counted. The graph displays the average number of Gal-3-positive puncta ± SD from three independent experiments. In total, 61–66 cells per group were analyzed. A one-way ANOVA was performed to assess variance, and comparisons were performed using Tukey’s post-hoc analysis. **p<0.01, ****p<0.0001.

**Fig. 3:**
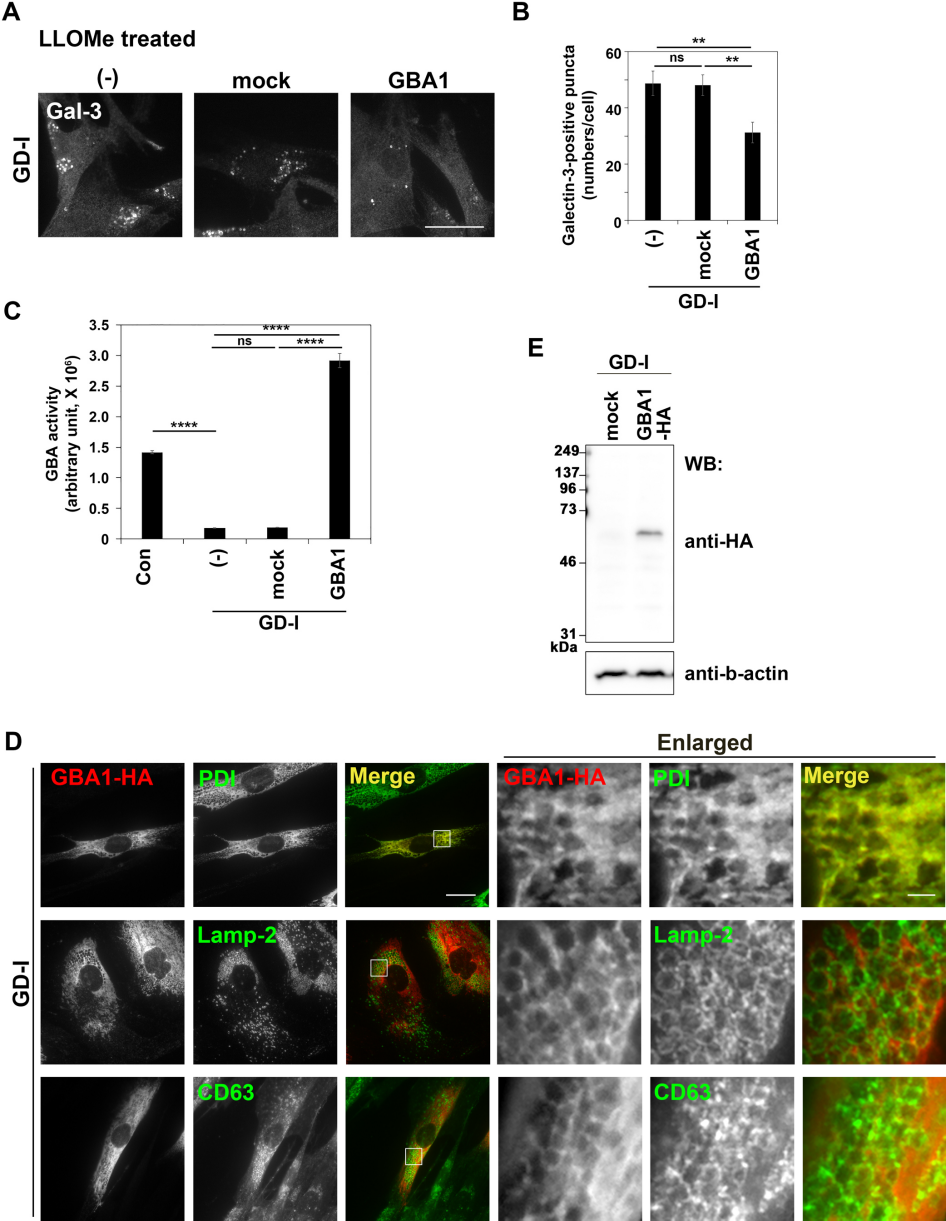
Exogenous expression of GBA1 can rescue the lysosomal integrity (A) GD-I fibroblasts (–), and GD-I fibroblasts stably expressing EGFP (mock) and C-terminally HA-tagged GBA1 were treated with LLOMe (0.5 mM), fixed, and stained for Gal-3. Bar, 40 μm. (B) The number of Gal-3-positive puncta was counted. In total, >100 cells per group were analyzed. The presented data represents a typical result from two independent experiments. The graph displays the average number of Gal-3-positive puncta ± SEM from all counted cells. Variance assessment was conducted through a one-way ANOVA, followed by Tukey’s post-hoc analysis for comparisons. **p<0.01, ns, not significant. (C) GD-I fibroblasts stably expressing EGFP (mock) and C-terminally HA-tagged GBA1 were lysed and GBA activity was measured. GBA activity was normalized to the protein concentration of each cell lysate. The presented data a representative of two independent experiments and the graph represents the mean ± SD of triplicate samples. Variance analysis was performed through a one-way ANOVA, followed by Tukey’s post-hoc analysis for comparisons. ****p<0.0001. ns, not significant. (D) GD-I fibroblasts stably expressing EGFP (mock) and C-terminally HA-tagged GBA1 were fixed, permeabilized, and incubated with anti-HA and anti-PDI (marker for the endoplasmic reticulum), anti-Lamp-2, or anti-CD63 antibodies followed by Cy3-conjugated anti-rat and Alexa Fluor 488-conjugated anti-mouse secondary antibodies. Bar, 20 μm. The boxed regions are enlarged and shown on the right side (Bar, 2 μm). (E) Expression levels of HA-tagged GBA1 were analyzed by immunoblotting with anti-HA and anti-β-actin (as an internal control) antibodies.

Next, we investigated lysosomal membrane integrity in GD cells. To this end, we treated cells with a lysosomotropic agent, l-leucyl-l-leucine methyl ester (LLOMe), and investigated lysosomal membrane permeability by immunostaining for a cytosolic lectin protein, Galectin-3 (Gal-3). Gal-3 is a β-galactose-binding lectin and a marker of damaged endosomes and lysosomes (Paz *et al.*, 2010). When the luminal sugar chain of lysosomes becomes accessible to cytosolic Gal-3, Gal-3 appears in puncta in cytoplasm. In the absence of LLOMe, Gal-3 was distributed throughout the cytoplasm and did not appear in puncta in both control and GD cells (Fig. 2A), indicating that lysosomes of GD cells are intact at steady state. A few Gal-3 puncta appeared in control cells upon treatment with 0.5 mM LLOMe for 15 min. By contrast, LLOMe treatment greatly increased the level of Gal-3 puncta in GD fibroblasts (Fig. 2B). Quantification of the number of Gal-3 puncta revealed a large increase of these puncta in GD cells (Fig. 2C). These results indicate that treatment with the lysosomotropic agent more readily damages lysosomal membranes of GD cells than those of control cells. To confirm the phenotype is due to the defect in GBA1, we established GD-I cells with the stable expression of wild-type GBA1 and subjected them to LLOMe treatment. The elevated number of Gal-3 puncta in GD-I cells was partially restored by the expression of GBA1 (Fig. 3A and B), indicating that the susceptibility of lysosomal membranes to LLOMe in GD-I cells was due to the mutation in the *GBA1* gene. As expected, GBA activity dramatically decreased in the GD-I cells as compared to control fibroblasts (Fig. 3C). GBA activity largely increased in GBA1 expressing GD-I cells but not in mock GD-I cells (Fig. 3C). The expression of GBA1 was confirmed by immunofluorescence and immunoblot analyses (Fig. 3D and E). It is worth noting that the majority of GBA1-HA was observed to localize to the endoplasmic reticulum (ER), and only a minor proportion was overlapped with Lamp-2 and CD63 (Fig. 3D), even if the expression of GBA1-HA could rescue the susceptibility of lysosomal membranes of GD-I cells (Fig. 3B). The ER-localization of exogenous GBA1-HA is likely a result of insufficient accessory proteins (e.g. LIMP-2, a receptor for GBA1) (Reczek *et al.*, 2007; Rothaug *et al.*, 2014) to facilitate the transport of the excessive amount of GBA1-HA to lysosomes. Indeed, exogenous GBA1 localizes to lysosomes rather than to the ER in *GBA1*-KO neuroblastoma cells (Navarro-Romero *et al.*, 2022).

### The cellular GlcCer level appears to vary among fibroblasts

**Fig. 4:**
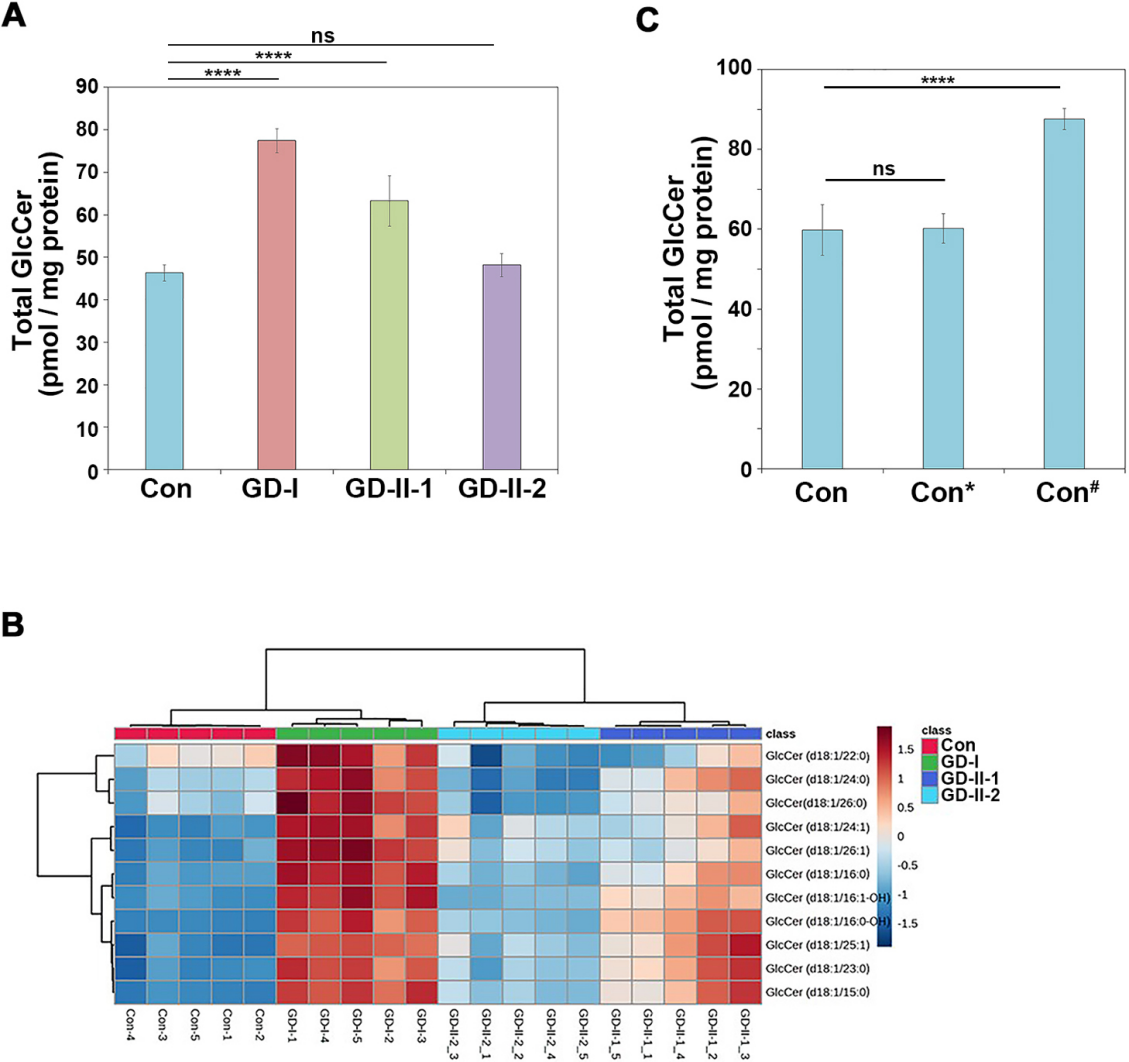
Cellular GlcCer levels in GD-derived fibroblasts Total lipids were extracted from normal (Con) and GD fibroblasts, and lipidomic analysis was performed. (A) The relative amount of total GlcCer detected. Data represent mean ± SD (quintuplicates of each sample). A one-way ANOVA was performed to assess variance, and comparisons with control fibroblasts were performed using Tukey’s post-hoc analysis. ****p<0.0001. ns, non-significant. (B) Hierarchical clustering heatmaps of GlcCer levels in fibroblasts. Pearson’s correlation analysis was performed for distance measure. Ward method, as a hierarchical clustering method, was used to create groups. (C) Total lipids were extracted from three different healthy (Con, Con*, and Con^#^) fibroblasts, and lipidomic analysis was performed. The relative amount of total GlcCer was determined. Data represent mean ± SD (quintuplicate of each sample). A one-way ANOVA was performed to assess variance, and comparisons with control fibroblasts were performed using Tukey’s post-hoc analysis. ****p<0.0001. ns, not significant.

We next examined the cellular GlcCer levels in healthy fibroblasts (Con) and the three GD fibroblasts by performing lipidomic analysis. The extracted ion chromatograms of GlcCer are shown in Supplementary Fig. S1. Although we could not discriminate between GlcCer and galactosylceramide in the lipidomic analysis, the majority of hexosylceramide in fibroblasts is predicted to be GlcCer, as galactosylceramide is not detected in human fibroblasts (Zama *et al.*, 2009). Therefore, we described GlcCer instead of hexosylceramide when referring to the data of the lipidomic analysis in this manuscript. The analysis is limited by the fact that the detected GlcCer levels are relative rather than absolute. The total amount of cellular GlcCer was found to be significantly higher in GD-I and GD-II-1 cells, but not in GD-II-2 cells, than in control cells (Fig. 4A). Fig. 4B shows the levels of individual GlcCer species in control and GD fibroblasts. Notably, we recognized that the intracellular GlcCer levels varied among three distinct healthy fibroblasts, with one exhibiting a higher level than the other two, implying the possible variations in the cellular GlcCer level among individuals (Fig. 4C). Thus, it remains uncertain whether the cellular GlcCer level is elevated in GD fibroblasts relative to control cells. However, it cannot be ruled out that the lysosomal GlcCer levels were increased in all GD fibroblasts, as we analyzed the GlcCer levels in whole cells.

## Discussion

This study examined the defects in fibroblasts derived from three GD patients with different mutations. Given that GD is the most common LSD characterized by the accumulation of GlcCer in lysosomes, it was anticipated that lysosomal functions would be affected. However, it was observed that lysosomal morphology and distribution remained largely unaltered in GD fibroblasts. Furthermore, lysosomal function, at least the protein degradation ability, was found to be unaffected. Therefore, the accumulation of GlcCer in lysosomes of GD fibroblasts may not disrupt lysosomal function. By contrast, lysosomal membranes of GD fibroblasts were more sensitive to the lysosomotropic agent LLOMe than those of control fibroblasts. Lysosomal membranes can be impaired by a variety of substances including mineral crystals, bacterial and viral toxins, lipids, β-amyloid, and lysosomotropic compounds (Boya and Kroemer, 2008; Dehay *et al.*, 2010; Emmerson *et al.*, 1990; Kroemer and Jaattela, 2005). Thus, lysosomes of cells derived from patients with GD may be readily damaged by these substances. Permeabilization of the lysosomal membrane results in leakage of contents from the lysosomal lumen into the cytosol, which can lead to apoptotic or necrotic cell death (Boya and Kroemer, 2008; Wang *et al.*, 2018). Macrophages are specialized cells that engulf many extracellular substances and thus may be markedly affected in patients with GD (Ferraz *et al.*, 2014; Stirnemann *et al.*, 2017). Lysosomal damage also activates the NLRP3 inflammasome, which induces secretion of proinflammatory cytokines, including interleukin-1β, which promote inflammation and enhance the pathogenesis of neurodegenerative disorders and LSDs (Bosch and Kielian, 2015; Hornung *et al.*, 2008; Ono *et al.*, 2018; Rigante *et al.*, 2017). The susceptibility of lysosomal membranes to damage in GD cells may be a pathogenic factor, as *GBA1*-deficient dendritic cells stimulate surrounding immune cells by releasing GlcCer through cell death or other mechanisms (Nagata *et al.*, 2017; Shimizu *et al.*, 2023). Indeed, the expression of wild-type GBA1 in GD fibroblasts can alleviate the vulnerability of lysosomal membranes, although only a minor proportion of GBA1 was found to localize to lysosomes.

How does the accumulation of GlcCer in lysosomes affect membrane integrity? It has been reported that an increase in the GlcCer levels alters the biophysical properties of artificial giant unilamellar vesicles (Varela *et al.*, 2016, 2017) and affects the structure and activity of toll-like receptor 4, which is activated by lipopolysaccharide (Mobarak *et al.*, 2018). Moreover, fibroblasts treated with conduritol-B-epoxide, which causes intracellular GlcCer accumulation, and fibroblasts from type I GD patients exhibit decreased membrane fluidity (Varela *et al.*, 2017). Thus, the accumulation of GlcCer or its metabolite, such as glucosylsphingosine (Dekker *et al.*, 2011), in cellular membranes, including lysosomes, can alter the biophysical properties of these membranes and affect their integrity.

Conflicting results have been reported regarding the increase in intracellular GlcCer in GD fibroblasts. Some studies have indicated that the cellular GlcCer levels are increased in GD fibroblasts (Fuller *et al.*, 2008; Zama *et al.*, 2009). However, other studies have reported that the GlcCer levels remain unchanged (Saito and Rosenberg, 1985; Sasagasako *et al.*, 1994; Sun *et al.*, 2015). Our lipidomic analysis revealed that the intracellular levels of GlcCer were elevated in GD-I and GD-II-1 cells, but not in GD-II-2 cells (Fig. 4A). Notably, variations were observed even among distinct control cells (Fig. 4C). Therefore, our study suggests that the cellular GlcCer levels appear to vary among the patients, individuals, and cell types (Sun *et al.*, 2015).

There are two primary treatment approaches for GD: enzyme replacement therapy (ERT) and substrate reduction therapy (SRT) (Stirnemann *et al.*, 2017). The principle of ERT is to supply glucocerebrosidase to cells that lack it, particularly GD cells. SRT aims to reduce excess cellular GlcCer by employing GlcCer synthase inhibitors (Mistry *et al.*, 2017; Stirnemann *et al.*, 2017). Symptomatic treatments are required for patients with GD because symptoms vary from individual to individual (Cox and Schofield, 1997; Lachmann *et al.*, 2004; Mistry *et al.*, 2017). Moreover, GD is associated with other diseases, such as PD and multiple myeloma (Arends *et al.*, 2013; Do *et al.*, 2019; Mistry *et al.*, 2013; Sidransky *et al.*, 2009).

The defect in lysosomal trafficking of GBA1 mutants could be a causative factor for GD. For example, ER retention and proteasomal degradation of the GBA1 mutants could be a factor for GD symptoms (Ron and Horowitz, 2005; Navarro-Romero *et al.*, 2022) and inhibition of ER-associated degradation can reduce the degradation rate of a GBA1 mutant in GD fibroblasts (Tan *et al.*, 2014). Therefore, the consideration of new therapeutic targets, will facilitate advances in the treatment of GD with complex symptoms.

## Materials and Methods

### Antibodies and reagents

Monoclonal mouse anti-EEA1 (clone14), anti-Lamp-1 (H4A3) and anti-Lamp-2 (H4B4) antibodies were purchased from BD Biosciences (San Jose, CA, USA). An anti-Golgin97 antibody (CDF4) was purchased from Invitrogen (Waltham, MA, USA). An anti-CD63 antibody was purchased from Ancell (Bayport, MN, USA). An anti-PDI antibody (1D3) was purchased from Enzo Life Sciences (Farmingdale, NY, USA). Anti-Galectin-3 (B2C10) and anti-β-actin (C4) antibodies were purchased from Santa Cruz (Dallas, TX, USA); A monoclonal rat anti-HA antibody (3F10) was purchased from Roche Applied Science (Basel, Switzerland). An anti-Galectin-3 antibody (M3/38) was obtained from Santa Cruz. Alexa Fluor-conjugated secondary antibodies were purchased from Invitrogen. Cy3- and horseradish peroxidase-conjugated secondary antibodies were purchased from Jackson ImmunoResearch Laboratories (West Grove, PA, USA). The EquiSPLASH Lipidomix quantitative standard for mass spectrometry, which includes ceramide (d18:1/15:0-d7) as an internal standard, was obtained from Avanti Polar Lipids (Ablabaster, AL, USA). The Self-Quenched BSA was purchased from BioVision (Waltham, MA, USA). BafA1 was purchased from LKT laboratories (St. Paul, MN, USA). Solvents such as methanol, isopropanol, and chloroform of LC-MS grade were purchased from Wako Pure Chemical Industries Ltd. (Osaka, Japan). Ammonium acetate and LLOMe were purchased from Sigma Aldrich (St. Louis, MO, USA).

### Plasmids

A cDNA encoding full-length GBA1 was obtained by PCR using a sense primer (5'-gccagatctaccatggagttttcaagtccttccag-3') and an antisense primer (5'-ccgctcgaggcctggcgacgccacaggtagg-3'). The cDNA obtained was subjected to PCR amplification using an additional forward primer (5'-gccagatctgccaccatggagttttcaagtccttccag-3') and the reverse primer incorporating Kozak consensus sequences. The amplified cDNA was inserted into the pENTR3C vector (Invitrogen), and the gene was transferred to pMXs-neo-DEST-HA using the Gateway system (Invitrogen) as described previously (Takatsu *et al.*, 2014).

### Cell culture and establishment of stable cell lines

Fibroblasts obtained from patients with type I GD (GM10915, L444P/L444P; designated GD-I), type II GD (GM01260, L444P/P415R, designated GD-II-1; and GM08760, L444P;E326K/L444P;E326K, designated GD-II-2) (Sun *et al.*, 2015), and three healthy individuals (GM00498, GM05757, and GM05659, designated Con, Con*, and Con^#^, respectively), were obtained from the Coriell Institute for Medical Research (Camden, NJ, USA). These fibroblasts were cultured in minimum essential medium (MEM; Nacalai Tesque, Kyoto, Japan) supplemented with MEM non-essential amino acids (Nacalai Tesque) and 10% fetal calf serum (Gibco, Waltham, MA, USA). Fibroblasts with stable expression of C-terminally HA-tagged GBA1, were established according to the previously described methods (Takatsu *et al.*, 2014).

### Immunofluorescence and Immunoblot analyses

For the lysosomal permeabilization assay, cells were treated with 0.5 mM LLOMe at 37°C for 15 min, then fixed, immunostained as described previously (Shin *et al.*, 2004), and observed using an Axiovert 200MAT microscope (Carl Zeiss, Thornwood, NY, USA). Immunoblot analysis was conducted as previously described (Takatsu *et al.*, 2011). Cells were lysed in lysis buffer (20 mM HEPES (pH 7.4), 150 mM NaCl, 1 mM EDTA, and 1% Nonidet P-40) containing a protease inhibitor mixture (Nacalai Tesque) at 4°C for 30 min. The lysates were centrifuged at maximum speed for 20 min at 4°C in a microcentrifuge to remove cellular debris. The lysates were incubated in SDS sample buffer including β-mercaptoethanol at 37°C for 2 h and subjected to SDS-PAGE and immunoblot analysis using rat anti-HA or mouse anti-β-actin antibody. Immunoblots were developed using ImmunoStar reagents (Wako) and recorded on an ImageQuant 800 (GE Healthcare, Chicago, IL, USA).

### Self-Quenched BODIPY-BSA treatment

Cells were pretreated with 100 nM BafA1 or an equivalent volume of DMSO in complete medium at 37°C for 1 h and then further incubated in medium containing 0.5% fetal calf serum, either 100 nM BafA1 or DMSO, and 10 μg/mL BODIPY-BSA at 37°C for 3 h. Cells were washed twice with ice-cold PBS (–) and incubated with ice-cold PBS (–) containing 5 mM EDTA and 0.5 μg/mL propidium iodide on ice for 30 min. Detached cells (more than 10^4^ cells/sample) were analyzed with a FACSCalibur instrument (BD Biosciences) to measure the fluorescence of dequenched BODIPY.

### GBA activity assay

The experiment was performed using the Glucosylceramidase Activity Assay Kit (ab273339, Abcam, Cambridge, UK) in accordance with the instruction manual. Fibroblasts (confluent in 100mm dish) were harvested and lysed with 200 μl of the Assay buffer XXV (containing 1% NP-40) on ice. Although the composition of the Assay buffer XXV was not disclosed, we verified that the buffer pH is approximately 5. Cell lysates were centrifuged at 14,500 rpm for 20 min at 4°C to remove cell debris. 20 μl of the lysates were mixed with the 1:20 diluted fluorescent substrate and incubated for 30 min at 37°C in 96 well plate (OptiPlate-96, Perkin Elmer, Waltham, MA, USA). After terminating the reaction by adding the Stop solution, the products were measured using EnVision microplate reader (Perkin Elmer) at Ex = 355 nm/Em = 460 nm. Protein concentration of the lysates was measured in parallel using protein assay CBB solution (Nacalai Tesque). The fluorescence was normalized with protein concentration.

### Liquid chromatography/mass spectrometry (LC/MS) analysis

A total of 5–6 ceramic beads (1.4 mm, Cat. No. 15-340-159, Fisherbrand, Pittsburgh, PA, USA) and 100 μL methanol were added to a frozen cell pellet in a 1.5 mL Eppendorf tube and homogenized using a BeadMill 4 homogenizer (Fisherbrand) for 30 seconds with two cycles. Total lipids were extracted from homogenates using the Folch method with modifications established previously in our laboratory (B. Gowda *et al.*, 2021; Folch *et al.*, 1957). In brief, 100 μL of an internal standard solution containing 18.8 μM ceramide (d18:1/15:0 (d7) in methanol was added to 100 μL methanolic homogenate. The mixture and vortexed at 3500 rpm for 30 s. Then, 400 μL chloroform and 100 μL milli-Q were added, followed by vortexing for 5 min. Extracts were centrifuged at 15,000 rpm for 10 min at 4°C, and the organic layer was transferred to a new Eppendorf tube. The aqueous layer was re-extracted with additional 400 μL chloroform. The combined chloroform extracts were evaporated and 100 μL methanol was added to redissolve lipid residues. About 10 μL sample was injected into the LC-MS system.

The analysis was conducted using a Prominence HPLC system (Shimadzu Corp., Kyoto, Japan) coupled with an LTQ Orbitrap mass spectrometer (Thermo Fisher Scientific Inc., San Jose, CA, USA). Chromatographic separation was achieved using a reverse-phase Atlantis T3 C18 column (2.1 × 150 mm, 3 μm; Waters, Milford, MA, USA) with an oven temperature set at 40°C. The mobile phases were (A) milli-Q containing 10 mM CH_3_COONH_4_, (B) isopropanol, and (C) methanol. A linear gradient was used as follows: 0–1 min, 30% B and 35% C; 1–14 min, 80% B and 10% C; 14–27 min, 85% B and 10% C; and 27–28 min, 30% B and 35% C. High-resolution mass spectrometry data were acquired in Fourier transform mode with a resolving power of 60,000, a collision energy of 35 eV, and a scan range of *m*/*z* 160–1900. Data-dependent acquisitions with tandem mass spectrometry were performed in ion-trap mode at a precursor ion isolation width of 3 *m*/*z* units and a collision energy of 40 eV. Quantification was performed after integrating the peaks using Xcalibur 2.2 software (Thermo-Fisher Scientific Inc.), according to the guidelines of Lipidomics Standards Initiative level 3 (https://lipidomics-standards-initiative.org/). Relative levels after normalizing the peak area by the protein content were calculated by determining the peak area ratios of the analytes to the internal standard and multiplying the peak area ratios using the amount of the added internal standard.

## Supplementary Material

Supplementary Fig. S1. Extracted ion chromatograms of GlcCer measured in fibroblasts by LC/MS
