# Supplementary figures and images for: Lysosomal membrane integrity in fibroblasts derived from patients with Gaucher disease
Source: Cell Struct Funct. 2023 Dec 9;49(1):1–10. doi: 10.1247/csf.23066 (PMC11496783; doi:10.1247/csf.23066)

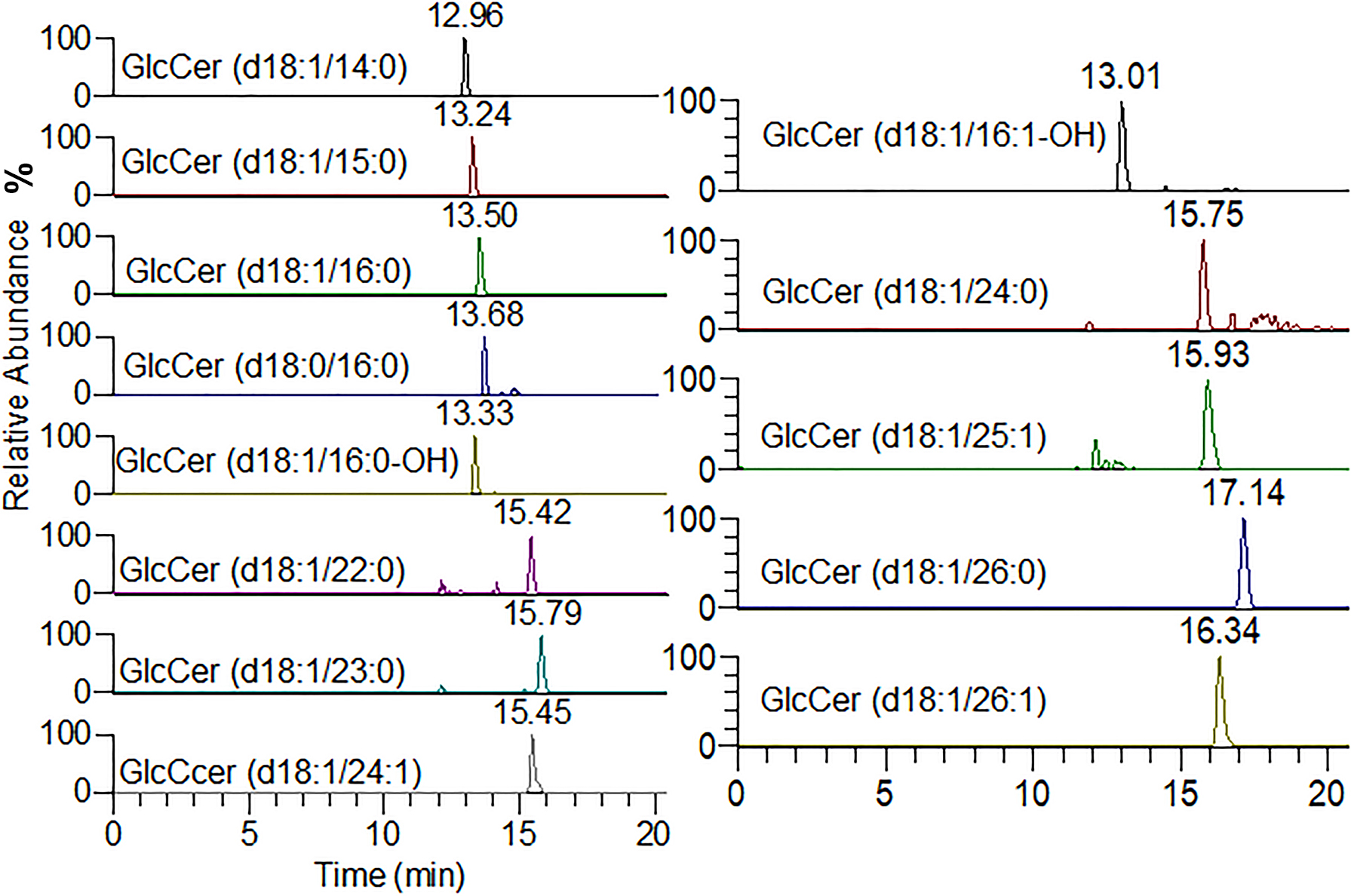

Supplement: Supplementary file 1 — Supplementary Fig. S1. Extracted ion chromatograms of GlcCer measured in fibroblasts by LC/MS [file csf_49_23066_1.png]
